# Supplementary figures and images for: Imidacloprid disrupts larval molting regulation and nutrient energy metabolism, causing developmental delay in honey bee Apis mellifera
Source: eLife. 2024 Mar 11;12:RP88772. doi: 10.7554/eLife.88772 (PMC10928512; doi:10.7554/eLife.88772)

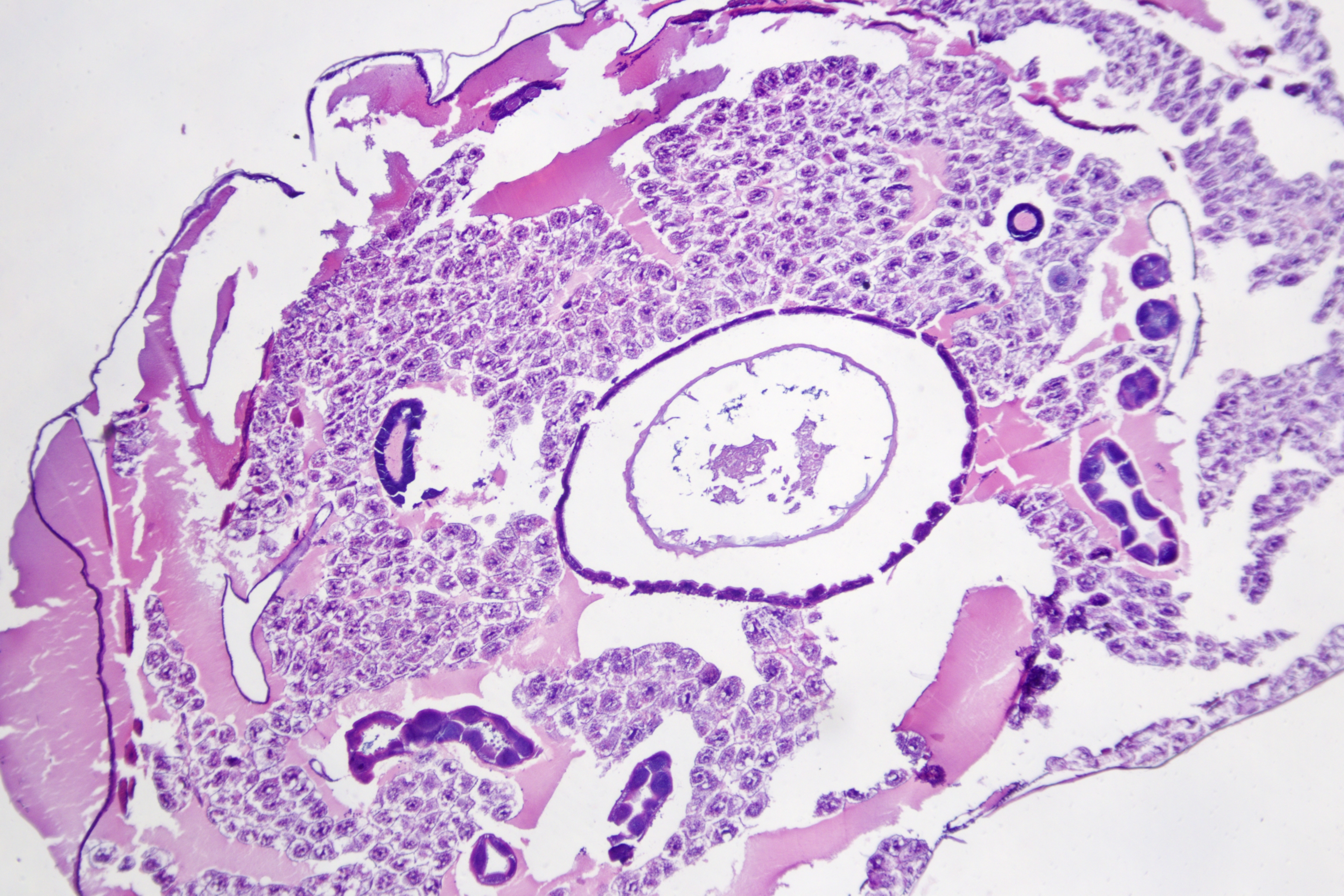

Supplement: Figure 5—source data 4. [file elife-88772-fig5-data4.zip › Figure 5D(CK)-source data .jpg]

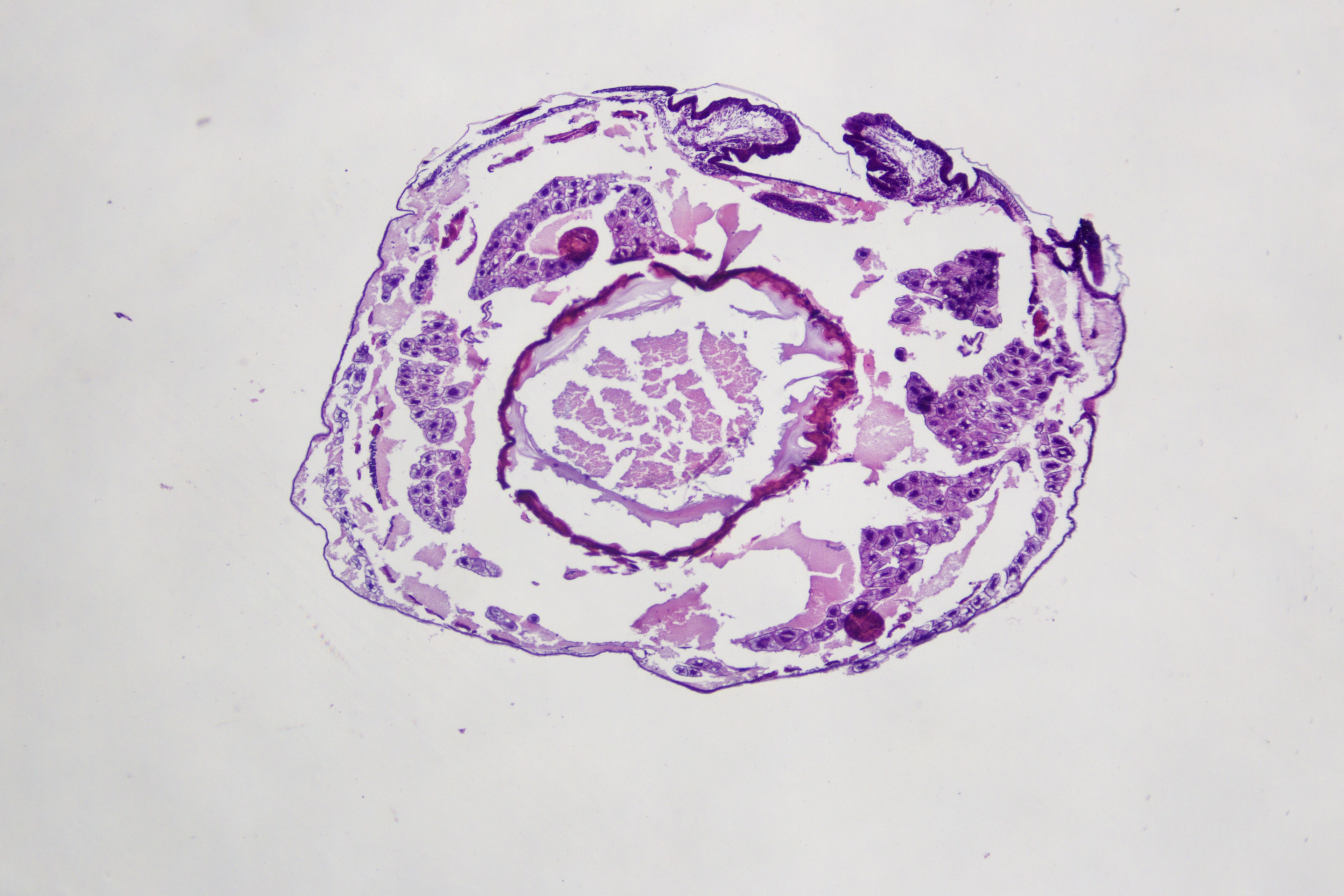

Supplement: Figure 5—source data 5. [file elife-88772-fig5-data5.zip › Figure 5D(IMI)-source data .jpg]
